# Financial protection effects of private health insurance: experimental evidence from Chinese households with resident basic medical insurance

**DOI:** 10.1186/s12939-021-01468-5

**Published:** 2021-05-17

**Authors:** Xian-zhi Fu

**Affiliations:** School of Political Science and Public Administration, Wuhan University, Wuhan, Hubei 430072 China

**Keywords:** Catastrophic health expenditure, Impoverishment, Medical insurance, Propensity score, China

## Abstract

**Background:**

After achieving universal basic medical insurance coverage, Chinese government put the development of private health insurance (PHI) on its agenda to further strengthen financial risk protection. This paper aims to assess the level of financial protection that PHI provides for its insured households on the basis of resident basic medical insurance (RBMI).

**Methods:**

We employed balanced panel data collected between 2015 and 2017 from the China Household Finance Survey (CHFS). Catastrophic health expenditure (CHE) and impoverishment due to health spending were applied to measure the financial protection effects. Random effects panel logistic regression model was performed to identify the factors associated with CHE and impoverishment among households covered by RBMI. In the robustness test, the method of propensity score matching (PSM) was employed to solve the problem of endogeneity.

**Results:**

From 2015 to 2017, the CHE incidence increased from 12.96 to 14.68 % for all sampled households, while the impoverishment rate decreased slightly from 5.43 to 5.32 % for all sampled households. In 2015, the CHE incidence and impoverishment rate under RBMI + PHI were 4.53 and 0.72 %, respectively, which were lower than those under RBMI alone. A similar phenomenon was observed in 2017. Regression analysis also showed that the households with RBMI + PHI were significantly less likely to experience CHE (marginal effect: -0.054, 95 %CI: -0.075 to -0.034) and impoverishment (marginal effect: -0.049, 95 %CI: -0.069 to -0.028) compared to those with RBMI alone. The results were still robust after using PSM method to eliminate the effects of self-selection on the estimation results.

**Conclusions:**

In the context of universal basic medical insurance coverage, the CHE incidence and impoverishment rate of Chinese households with RBMI were still considerably high in 2015 and 2017. PHI played a positive role in decreasing household financial risk on the basis of RBMI.

**Supplementary Information:**

The online version contains supplementary material available at 10.1186/s12939-021-01468-5.

## Background

One of the fundamental functions of a healthcare system is to establish a health financing system that protects households from illness-associated financial risks [1]. This is mainly because the existence of financial risks inevitably creates some difficulties in demand-oriented access to health care.

As the world’s largest developing economy, China has implemented a series of health system reforms in response to the challenge of financial risks from diseases. In 2009, China launched a new round of health system reform to achieve the goal of provision of affordable and equitable essential health care by 2020 [2, 3]. As a result of this comprehensive reform, the coverage rate of basic medical insurance has increased significantly. In 2011, a universal medical insurance system was basically formed, with more than 1.3 billion individuals covered by three major programs [4]: Urban Employee Basic Medical Insurance (UEBMI) designed for urban workers, implemented in 1998; New Rural Cooperative Medical Scheme (NRCMS) designed for rural residents, established in 2003; and Urban Resident Basic Medical Insurance (URBMI) designed for urban residents not covered by UEBMI, initiated in 2007 [5–7]. In some areas of mainland China, URBMI and NRCMS have been integrated into the urban-rural resident basic medical insurance (URRBMI) to eliminate the disparity between urban and rural residents in terms of health service utilization [8]. The establishment of basic medical insurance could solve the problem of " “poverty caused by illness” to a certain extent, but some scholars have confirmed that its ability to protect against financial risk is limited [9–11]. According to official data from the National Health and Family Planning Commission of China, in 2013, approximately 12.56 million households in China were pushed into poverty due to medical costs, accounting for 42.4 % of all poor households [12]. This is mainly because the basic medical insurance follows the principle of “low level and wide coverage”. In detail, the basic medical insurance coverage formulated by the Chinese healthcare security department is very broad, involving specific drug catalogs, diagnosis and treatment items, and the scope of medical service facilities. According to the reimbursement rate set by the local government, the portion of medical expenses between the standard of deductible franchise and the annual limit to pay is reimbursed by the basic medical insurance. The setting of the ceiling line may lead to the illness-associated financial risks for households paying large medical bills.

Against the backdrop of universal basic medical insurance coverage, Chinese government put the development of PHI on its agenda to further improve protection for financial risk [13, 14]. At present, the development of PHI in China is still in its initial stage. The fifth round of China’s National Health Service Survey showed that the coverage rate of PHI was only 6.9 % in 2013, roughly the same level as in 2008 [15]. In mainland China, a small number of cities (e.g., Hangzhou, Shaoxing, Taizhou, Wenzhou, Zhoushan, etc.) have released implementation plans for PHI [16–20]. As a supplementary form of medical insurance, PHI mainly reimburses patients for high out-of-pocket medical expenses after reimbursement by basic medical insurance. Specifically, PHI covers essential, effective and affordable items, including but not limited to the coverage of basic medical insurance. The implementation plans in some cities initially set the standard of deductible franchise, reimbursement rate and ceiling line for PHI [18–20], as detailed in Table 1. At this stage, it is a question worthy exploring whether PHI can further play an effective role in improving protection for financial risk on the basis of basic medical insurance. The answer to this question has important implications for improving China’s medical security system.

**Table 1:** Reimbursement policies for private health insurance in some cities, China

Cities	Year of publication	Standard of deductible franchise	Reimbursement rate	Ceiling line
Hangzhou	2020	<=20,000 CNY	>=70 %	>=1,000,000 CNY
Shaoxing	2021	-	>=50 %	>=1,500,000 CNY
Zhoushan	2021	-	>=50 %	>=1,500,000 CNY

Catastrophic health expenditure (CHE) and impoverishment due to health spending are common indicators used to measure the status of protection for financial risk [21–23]. Most of the existing literature in China has focused on assessing the trends in the incidences of CHE and impoverishment at the national or provincial level since the new round of health system reform [11, 24–27]. Some studies have focused on estimating the extent of CHE and impoverishment among vulnerable groups (e.g., patients with chronic diseases, rural residents, elderly) [10, 28–31]. Some studies have focused on exploring the effect of basic medical insurance on the incidences of CHE and impoverishment in China [9, 23, 32]. In general, the effects of PHI on financial catastrophe and impoverishment due to health spending in China at national level are still unclear.

In addition, some scholars have applied the idea of “experimental research” to assess the effect of related policies on the incidences of CHE and impoverishment. Wagstaff (2007) combined differences-in-differences (DID) with propensity score matching (PSM) to estimate the impact of a health reform project in China that combined supply-side interventions aimed at expanding medical insurance and providing financial support to the very poor [33]. Jeong (2012) verified the effect of the benefit expansion policy on CHE in Korea through experimental evidence [34]. Chen (2019) also verified the impact of the health poverty alleviation project on the incidences of CHE and impoverishment among rural residents in China through experimental evidence [35]. Introducing the idea of “experimental research” into this study may solve the problems of endogeneity to improve the credibility of the findings.

This study used a nationally representative household survey to assess the level of financial protection that PHI provided for its insured households on the basis of basic medical insurance. Given that the policy benefits provided by the resident basic medical insurance (RBMI) were far inferior to those provided by UEBMI [36, 37], we focused on the more vulnerable households, which were covered by RBMI (including NRCMS, URBMI and URRBMI). The specific objectives of this study are as follows: (1) to estimate the incidences of CHE and impoverishment due to health spending at the national level, stratified by different insured groups; (2) to verify the effect of PHI on CHE and impoverishment due to health spending on the basis of RBMI; and (3) to perform the robustness test. It should be emphasized that we solved the endogenous problems caused by self-selection in the robustness test.

## Methods

### Data source

This paper used two waves of data from the China Household Finance Survey (CHFS) conducted in 2015 and 2017. The CHFS is a nationally representative longitudinal tracking survey conducted by Southwestern University of Finance and Economics of China every two years from 2011 to 2017. Face-to-face household interviews were conducted by trained investigators using standardized questionnaires. The questionnaire involves detailed information on demography characteristics and socioeconomic status of households, health status, inpatient service utilization and medical insurance of household members.

A robust three-stage (districts/counties-villages/communities-households), stratified, random sampling method was employed to ensure representation of the whole population in China. In the first stage, 351 counties (districts) in 2015 and 355 counties (districts) in 2017 were randomly selected from 29 provinces (excluding Xinjiang, Tibet) in mainland China. In the second stage, 1396 villages (communities) in 2015 and 1428 villages (communities) in 2017 were selected in sampled counties or districts. In the third stage, 37,289 households in 2015 and 40,011 households in 2017 were selected in sampled villages or communities. After excluding the households with missing variables and/or with logic error answers, 27,080 households in 2015 and 39,988 households in 2017 were left. After excluding the households not covered by RBMI (including NRCMS, URBMI and URRBMI), 21,867 households in 2015 and 31,137 households in 2017 were left. According to the household ID, we matched the households in the two waves of the survey to obtain a balanced panel data. Finally, 18,149 valid households from the two waves of the survey, including 36,298 observations, were adopted for the empirical analysis.

It is worth emphasizing that the household ID in two waves of the survey are mapped to the original households one by one. Specifically, if all members of an original household have moved to elsewhere, the investigator needs to follow up with the original household based on the latest contact information or contact address. Households at the original address are defined as original households as long as there are household members living at the original address.

### Definitions of dependent variables

The approach reported by Wagstaff and van Doorslaer was employed to measure CHE [38]. We set out-of-pocket medical expenses as the numerator and household’s capacity to pay as the denominator to calculate the CHE incurrence. It should be noted that indirect expenditures (e.g., accommodation cost, food, lost productivity due to illness, etc.) were not included in the out-of-pocket medical expenses due to the unavailability of data. Since the substitution of non-food household expenditure for total household expenditure partly avoided the measurement deviations that were often overlooked in poor households, we set the non-food household expenditure as an indicator to measure the capacity to pay of a household [28, 29]. Thus, health expenditure was defined as catastrophic when out-of-pocket medical expenditure was equal to or higher than a given threshold of a household’s non-food expenditure. Following previous studies, the threshold for CHE was defined as 40 % [39, 40].

High out-of-pocket medical expenses may push a non-poor household into poverty. In this study, a household was considered impoverished when its per capita household consumption expenditure was equal to or above the poverty line, but below the poverty line net of per capita out-of-pocket medical expenses [22, 32]. We used the poverty line of 1.90 US$ per person per day developed by the World Bank, which was commonly referred to as the extreme poverty line [41]. After adjustment of the 2014 exchange rate (i.e. 1 US$ = 6.14 CNY), the poverty line in 2015 was equal to 4258.09 CNY per year [42]. As per capita household consumption expenditure in the year 2017 was deflated to the year of 2015 by using consumer price index, the poverty line was set as the same level for both years.

### Definitions of independent variables and control variables

Medical insurance was the independent variable in this study. In addition, with reference to the existing studies [24–26, 28, 29, 43, 44], control variables included a series of variables related to the characteristics of each household and its household head. Households characteristics involved seven variables: household size, receiving inpatient services, having members below 5 years old, having members over 60 years old, economic status, geographic location, and residency location. The characteristics of household head included five variables: gender, education, marital status, employment status, and self-rated health status.

The annual household income per capita was employed to measure the economic status of a household. Table 2 presents the detailed description of the above independent variables and control variables.

**Table 2:** Description of independent variables and control variables

Variables	Description
Independent variables	
Medical insurance	At least one household member covered by PHI; RBMI + PHI = 1; RBMI alone = 0.
Control variables	
Household size	The number of household members.
Inpatient	At least one household member received inpatient services in last year; Yes = 1; No = 0.
Household members aged < = 5	At least one household member below 5 years old; Yes = 1; No = 0.
Household members aged > = 60	At least one household member over 60 years old; Yes = 1; No = 0.
Economic status (CNY)	The annual household income per capita.
Geographic location	East = 1; Central = 2; West = 3.
Residency location	Urban = 1; Rural = 0.
Gender of household head	Male = 1; Female = 0.
Education of household head	Illiterate = 1; Primary school = 2; Middle school = 3; High school and above = 4.
Marriage of household head	Married = 1; Unmarried = 0.
Employment status of household head	Unemployed and others = 1; Employed = 2; Retired = 3.
Self-rated health of household head	Healthy = 1; Unhealthy = 0.

Note: *RBMI* Resident basic medical insurance; *PHI* Private health insurance; Annual household income per capita in the year 2017 was deflated to the year of 2015 by using consumer price index

### Methodology

Given the incidences of CHE and impoverishment were binary outcome variables, logistic regression model was applied to analyze the impact of PHI on the incidences of CHE and impoverishment. Generally, panel logistic regression model can be categorized as fixed effects panel logistic regression model and random effects panel logistic regression model. Specifically, the fixed effects panel logistic regression model is chosen when individual heterogeneity is correlated with other independent variables, while the random effects panel logistic regression model is chosen when individual heterogeneity is independent of other independent variables. However, the fixed effects panel logistic regression model would be a poor choice in a situation where the independent variables don’t change much over time. In this study, most of the households interviewed include variables (e.g., geographic location, residency location, gender of the household head, etc.) that do not change over time. Given the strict households inclusion criteria for the fixed effects panel logistic regression model, we applied random effects panel logistic regression model to verify the effects of PHI on financial catastrophe and impoverishment due to health care spending. The model is as follows:

1 $$logit\left(\frac{{Y}_{it}}{{1-Y}_{it}}\right)={\beta }_{0}+{\beta }_{1}*{Medi}_{it}+{\beta }_{2}*{Z}_{it}+{\epsilon }_{it}\left({\epsilon }_{it}={\alpha }_{i}+{v}_{it}\right)$$

In the Eq. (1), $Y$ represents the probability of incurring CHE or impoverishment for household $i$ in the period $t$, $Medi$ indicates the medical insurance, $Z$ indicates the control variables, ${\beta }_{1}$ and ${\beta }_{2}$ are the marginal effect, and ${\epsilon }_{it}$ denotes the error term. The error term is composed of two parts, ${\alpha }_{i}$ and ${v}_{it}$, which denote the unobservable random variables (individual heterogeneity) and the independently and identically distributed disturbance term, respectively. We hypothesize that ${\alpha }_{i}$ follows a logistic distribution, while ${v}_{it}$ follows an independently and identically distributed normal distribution. Given that the distribution of ${\epsilon }_{it}$ is difficult to determine, we hypothesize that it follows a logistic distribution.

To make the results more convincing, the method of PSM was adopted to perform a robustness test. As purchasing PHI is a self-selection behavior rather than a random distribution, it may lead to biased estimates. Specifically, a series of variables related to the characteristics of household and its household head may affect residents’ participation in PHI, and these variables may also affect the dependent variables. This led to endogeneity problems in estimating the impact of PHI on the dependent variables. Theoretically, the method of PSM is based on the propensity score to match the treatment group and the control group, so that the endowment difference between the two groups after matching is not statistically significant [45]. Therefore, the bias of estimation results could be excluded to some extent, and the net effect of PHI on alleviating financial risks associated with diseases could be obtained.

In the first stage, this study calculated the propensity score by a logistic regression model. The propensity score represents the conditional probability of the interviewed household entering the treatment group given a series of variables [45]. The equation is as follows:

2 $$logit\left(\frac{P}{1-P}\right)={\gamma }_{0}+{\gamma }_{1}*X+\mu$$

In the Eq. (2), $P$ denotes the likelihood of participating PHI for a household. ${\gamma }_{0}$, ${\gamma }_{1}$ and ${\upmu }$ represent the intercept term, regression coefficient and error term, respectively. $X$ are the covariates that affect a household’s purchase of PHI. After filtering these covariates using the STATA command “psestimate”, we set all control variables in Table 2 as covariates except for the marriage of household head.

In the second stage, according to the propensity score, each household with RBMI + PHI was matched with a household with RBMI alone. We performed one-to-one and nearest-neighbor matching with 0.25 times standard deviations of the propensity score as a caliper value.

In the third stage, the average treatment effect on the treated (ATT) was calculated based on the households from the treatment group and control group. The calculation of ATT can be specified as:

3 $$ATT=E\left({Y}_{1i}^{T}\right|{P}_{i}=1)-E({Y}_{0i}^{C}|{P}_{i}=0)$$

In the Eq. (3), $P$ represents the likelihood of participating PHI for household $i$, $Y$ denotes the probability of incurring CHE or impoverishment for household $i$, $C$ is the control group (RBMI alone), and $T$ indicates the treatment group (RBMI + PHI).

All statistical analyses were performed using STATA software version 16.0 and a *p*-value < 0.05 was regarded as statistically significant.

## Results

### Descriptive statistic

Table 3 summarizes the distribution of relevant characteristics of households. The proportion of households covered by RBMI + PHI rose from 2.31 % to 2015 to 7.71 % in 2017. A fluctuation was observed for the household size, which started from 3.975 to 2015 declined to 3.546 in 2017. The proportion of households receiving inpatient services and having children below 5 years old increased slightly, while the percentage of households having elderly members declined slightly. The annual household income per capita grew with survey year, from 15,174 CNY in 2015 to 20,646 CNY in 2017. Surveyed households in the eastern region are most prevalent, accounting for around 41.25 % of households across the two surveys, followed by the central region (around 30.87 %) and western region (around 27.88 %). Concerning household heads with education level of middle school, there was an increase from 35.69 % to 2015 to 36.26 % in 2017. More than 70 % of household heads were employed. The proportion of female household heads, married household heads, and healthy household heads declined slightly in the year of 2015 to 2017.

**Table 3:** Characteristics of households interviewed, China, 2015–2017

Variables	2015		2017	
	N	%	N	%
Medical insurance				
RBMI + PHI	419	2.31	1399	7.71
RBMI alone	17,730	97.69	16,750	92.29
Inpatient				
Yes	5453	30.05	5915	32.59
No	12,696	69.95	12,234	67.41
Household members aged < = 5				
Yes	2257	12.44	2987	16.46
No	15,892	87.56	15,162	83.54
Household members aged > = 60				
Yes	10,398	57.29	9858	54.32
No	7751	42.71	8291	45.68
Geographic location				
East	7487	41.25	7487	41.25
Central	5602	30.87	5602	30.87
West	5060	27.88	5060	27.88
Residency location				
Urban	9537	52.55	9559	52.67
Rural	8612	47.45	8590	47.33
Gender of household head				
Female	3221	17.75	2824	15.56
Male	14,928	82.25	15,325	84.44
Education of household head				
Illiterate	1731	9.54	1633	9.00
Primary school	5679	31.29	5610	30.91
Middle school	6477	35.69	6581	36.26
High school and above	4262	23.48	4325	23.83
Marriage of household head				
Married	16,316	89.90	16,108	88.75
Unmarried	1833	10.10	2041	11.25
Employment status of household head				
Unemployed and others	4285	23.61	4036	22.24
Employed	13,070	72.01	12,897	71.06
Retired	794	4.37	1216	6.70
Self-rated health of household head				
Healthy	14,599	80.44	14,133	77.87
Unhealthy	3550	19.56	4016	22.13
Observations	18,149	100.00	18,149	100.00

Note: *RBMI* Resident basic medical insurance; *PHI* Private health insurance

### Incidences of CHE and impoverishment

**Fig. 1:**
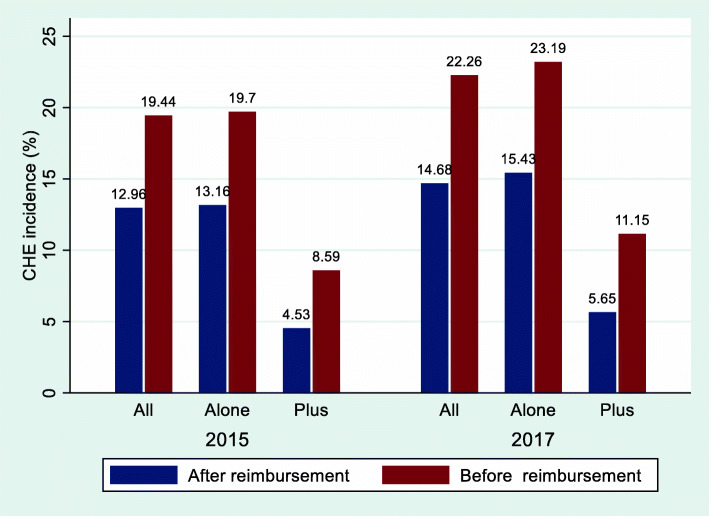
Incidence of CHE among all the households interviewed before and after reimbursement, China, 2015–2017. Notes: *All* indicates all the households interviewed; *Alone* indicates the households only covered by resident basic medical insurance; *Plus* indicates the households covered by resident basic medical insurance plus private health insurance

Figure 1 presents lower CHE incidence after reimbursement compared with before. The overall proportion of households incurring CHE increased from 12.96 % to 2015 to 14.68 % in 2017. In 2015 and 2017, the CHE incidences under RBMI + PHI were 4.53 and 5.65 %, respectively, which were lower than those under RBMI alone. After reimbursement from RBMI alone, the CHE incidence decreased by 33.2 % in 2015 and 33.46 % in 2017, respectively. Under RBMI + PHI, the CHE incidence decreased by 47.26 % in 2015 and 49.33 % in 2017, respectively.

**Fig. 2:**
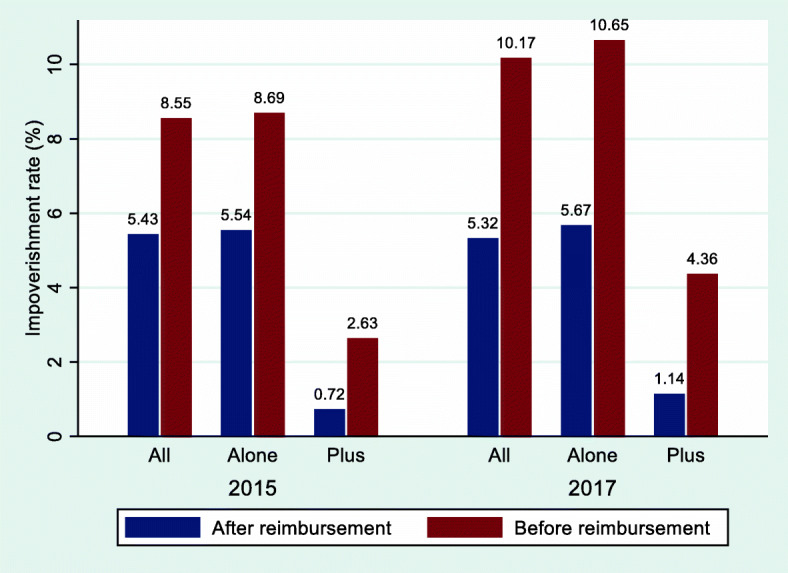
Rate of impoverishment among all the households interviewed before and after reimbursement, China, 2015–2017. Notes: *All* indicates all the households interviewed; *Alone* indicates the households only covered by resident basic medical insurance; *Plus* indicates the households covered by resident basic medical insurance plus private health insurance

As shown in Fig. 2, the impoverishment rate of all sampled households dropped slightly from 5.43 % to 2015 and 5.32 % in 2017. The impoverishment rate was less under RBMI + PHI than under RBMI alone (2015: 0.72 % vs. 5.54 %, 2017: 1.14 % vs. 5.67 %). After reimbursement from RBMI alone, the impoverishment rate decreased by 36.25 % in 2015 and 46.76 % in 2017, respectively. Under RBMI + PHI, the impoverishment rate decreased by 72.62 % in 2015 and 73.85 % in 2017, respectively.

### Associated factors of catastrophic health expenditure and impoverishment

Table 4 shows the results of random effects panel logistic regression model for CHE and impoverishment from health spending. After controlling a series of variables, households covered by RBMI + PHI were significantly less likely to experience CHE (marginal effect: -0.054, 95 %CI: -0.075 to -0.034) and impoverishment (marginal effect: -0.049, 95 %CI: -0.069 to -0.028) compared with households covered by RBMI alone.

**Table 4:** The factors associated with catastrophic health expenditure and impoverishment due to health spending, using logistic regression

Variables	Catastrophic health expenditure		Impoverishment due to health spending	
	dy/dx	95 %CI	dy/dx	95 %CI
Medical insurance (Ref: RBMI alone)	-0.054**	[-0.075, -0.034]	-0.049**	[-0.069, -0.028]
Household size	-0.021**	[-0.024, -0.019]	-0.003**	[-0.005, -0.002]
Inpatient (Ref: No)	0.163**	[0.157, 0.169]	0.051**	[0.046, 0.056]
Household members aged < = 5 (Ref: No)	0.008	[-0.003, 0.019]	0.006	[-0.001, 0.014]
Household members aged > = 60 (Ref: No)	0.047**	[0.040, 0.055]	0.021**	[0.015, 0.026]
Economic status	-0.007**	[-0.009, -0.006]	-0.005**	[-0.006, -0.004]
Geographic location (Ref: East)				
Central	0.010*	[0.002, 0.018]	0.012**	[0.006. 0.017]
West	-0.013**	[-0.021, -0.004]	0.006*	[0.001, 0.012]
Residency location (Ref: Urban)				
Rural	0.019**	[0.011, 0.026]	0.030**	[0.025, 0.035]
Gender of household head (Ref: Female)	0.008	[-0.001, 0.018]	0.008*	[0.001, 0.015]
Education of household head (Ref: Illiterate)				
Primary school	-0.019**	[-0.031, -0.007]	-0.008	[-0.016, 0.000]
Middle school	-0.033**	[-0.045, -0.020]	-0.018**	[-0.027, -0.010]
High school and above	-0.055**	[-0.068, -0.041]	-0.031**	[-0.040, -0.022]
Marriage of household head (Ref: Unmarried)	-0.002	[-0.013, 0.009]	0.015**	[0.007, 0.023]
Employment status of household head (Ref: Unemployed and others)				
Employed	-0.046**	[-0.055, -0.038]	-0.010**	[-0.015, -0.004]
Retired	-0.006	[-0.022, 0.010]	-0.033**	[-0.043, -0.024]
Self-rated health of household head (Ref: Unhealthy)	-0.070**	[-0.077, -0.063]	-0.029**	[-0.033, -0.024]

Note: *RBMI alone* indicates the households only covered by resident basic medical insurance; The dy/dx in brackets indicates the marginal effect; * *p* < 0.05; ** *p* < 0.01

With respect to control variables, household size and household economic status were negatively correlated with the incidences of CHE and impoverishment. Receiving inpatient services in the last 12 months was positively associated with the incidences of CHE and impoverishment. Having elderly members was positively correlated with the incidences of CHE and impoverishment. Households in rural areas were more prone to fall into CHE and impoverishment. Better self-rated health status and higher education attainment of household head significantly decreased the incidences of CHE and impoverishment. The risks of CHE and impoverishment were significantly decreased among households with an employed head, and the probability of incurring impoverishment was significantly decreased among households with a retired head.

### Robust test

**Fig. 3:**
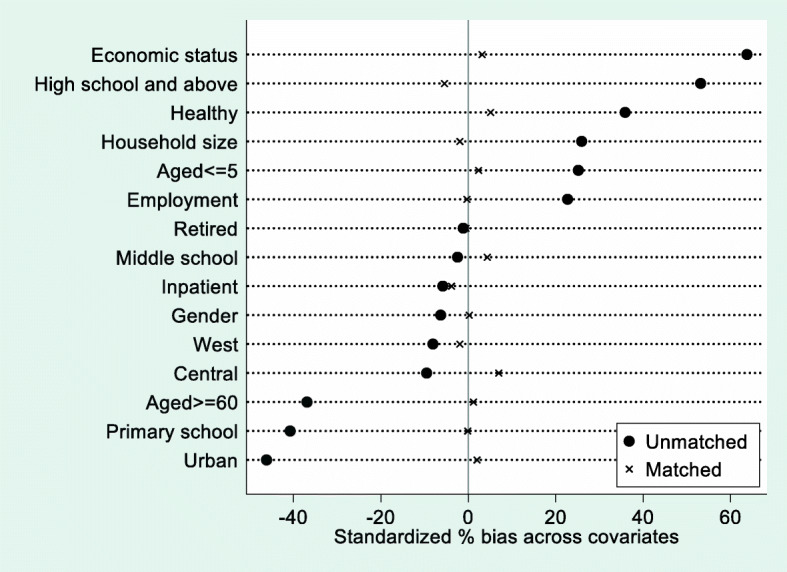
Standardized difference scatter plot of all covariates before and after propensity score matching (%)

**Fig. 4:**
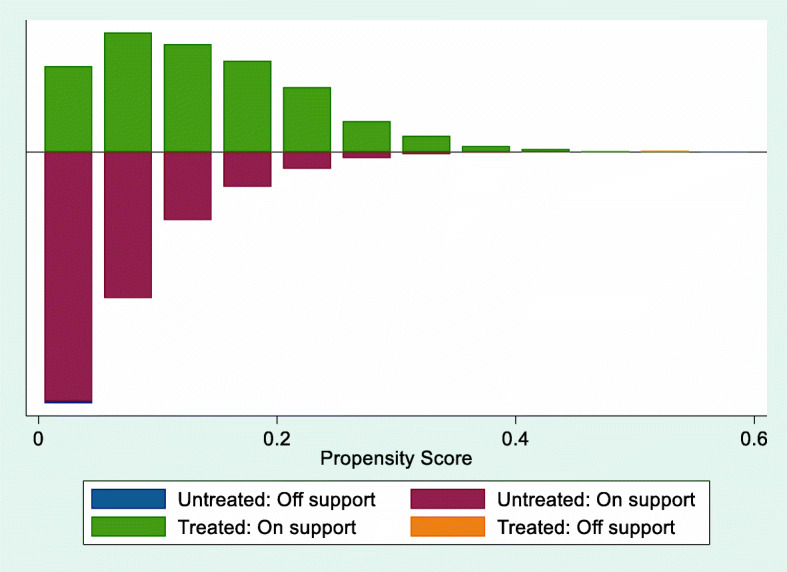
Distribution range of propensity score for the treatment group and the control group

In this section, we tested the robustness of the results by using PSM method. Figure 3 displays the changes of standardized bias of all covariates before and after propensity score matching. Figure 4 illustrates the distribution range of propensity score for treatment group and control group. Table 5 presents the detailed results of balanced test for the quality of propensity score matching.

**Table 5:** Balance test for the quality of propensity score matching in 2017

Variables		Mean		Bias (%)	Bias reduction (%)	T-test	
		Treated group	Control group			t-value	*P*>|t|
Household size	Unmatched	3.905	3.516	25.9		8.67	0.000
	Matched	3.905	3.934	-2.0	92.5	-0.53	0.599
Inpatient	Unmatched	0.301	0.328	-5.8		-2.08	0.038
	Matched	0.301	0.319	-3.9	33.9	-1.02	0.306
Household members aged < = 5	Unmatched	0.258	0.157	25.2		9.84	0.000
	Matched	0.258	0.248	2.3	90.8	0.57	0.572
Household members aged > = 60	Unmatched	0.376	0.557	-36.9		-13.13	0.000
	Matched	0.377	0.371	1.2	96.8	0.31	0.755
Economic status (CNY)	Unmatched	9.970	9.072	63.7		21.75	0.000
	Matched	9.965	9.921	3.1	95.1	0.93	0.354
Central	Unmatched	0.269	0.312	-9.5		-3.36	0.001
	Matched	0.269	0.238	6.9	27.2	1.91	0.056
West	Unmatched	0.246	0.282	-8.1		-2.86	0.004
	Matched	0.246	0.255	-2.0	75.9	-0.52	0.600
Urban	Unmatched	0.272	0.490	-46.2		-15.83	0.000
	Matched	0.272	0.263	2.0	95.7	0.56	0.579
Gender of household head	Unmatched	0.823	0.846	-6.3		-2.33	0.020
	Matched	0.822	0.822	0.2	97.0	0.05	0.961
Primary school	Unmatched	0.152	0.322	-40.8		-13.28	0.000
	Matched	0.152	0.153	-0.2	99.6	-0.05	0.958
Middle school	Unmatched	0.352	0.364	-2.5		-0.89	0.376
	Matched	0.352	0.331	4.3	-75.3	1.16	0.248
High school and above	Unmatched	0.463	0.220	53.2		20.79	0.000
	Matched	0.462	0.487	-5.5	89.7	-1.33	0.185
Employed	Unmatched	0.801	0.703	22.7		7.74	0.000
	Matched	0.800	0.802	-0.3	98.5	-0.09	0.925
Retired	Unmatched	0.064	0.067	-1.2		-0.42	0.678
	Matched	0.064	0.066	-0.6	50.5	-0.15	0.878
Healthy	Unmatched	0.900	0.769	35.9		11.41	0.000
	Matched	0.900	0.881	5.1	85.8	1.58	0.115

Most of the observed values were within the range of common support, which means that only a small number of households will be lost after the propensity score matching. The standardized bias of all covariates decreased significantly after propensity score matching. The absolute values of the standardized bias were less than 10 % for all covariates after matching, indicating that the matching quality was sufficiently high. The *p* values of the t-test also demonstrated that there was no significant difference in the distribution of relevant characteristics between the two groups.

Table 6 shows the average treatment effect on the treatment group. After matching, the incidences of CHE and impoverishment of the control group were significantly higher than those of the treatment group, and the ATT values of the two indicators were − 0.037 and − 0.015, respectively. These results indicated that PHI decreased the probability of incurring CHE and impoverishment by 3.7 and 1.5 %, respectively.

**Table 6:** The effect of private health insurance on catastrophic health expenditure and impoverishment due to health spending in 2017

Dependent variables		Treated group	Control group	ATT	S.E.	T-value
Catastrophic health expenditure	Unmatched	0.056	0.154	-0.098**	0.010	-9.96
	Matched	0.057	0.093	-0.037**	0.011	-3.35
Impoverishment due to health spending	Unmatched	0.011	0.057	-0.045**	0.006	-7.26
	Matched	0.011	0.026	-0.015**	0.006	-2.65

Note: *ATT* in brackets indicates the Average Treatment Effect on the Treated; * *p* < 0.05; ** *p* < 0.01

## Discussion

The introduction of PHI as a supplement to the basic medical insurance is an important step for the Chinese government to solve the problem of “poverty caused by illness” among urban and rural residents. However, the effect of PHI to address this problem has been neglected. To our best knowledge, this study is the first nationally representative study on the effects of PHI on financial catastrophe and impoverishment due to health spending in China. Using balanced panel data collected between 2015 and 2017 from the CHFS, this study estimates the incidences of CHE and impoverishment due to health spending for households with RBMI alone and RBMI + PHI, and assesses the level of financial protection that PHI provides for its insured households on the basis of RBMI.

After reimbursement, the CHE incidence and impoverishment rate of all sampled households were still considerably high in both years. These estimates were close to the results of some existing studies, which focused on Chinese households as a whole [24–26], and significantly higher than those found in other developing countries [46, 47]. We also found that households reimbursed from RBMI + PHI had lower incidences of CHE and impoverishment due to health spending than households reimbursed from RBMI alone, suggesting a lower economic risk for the former. Two potential reasons could be responsible for this phenomenon. Firstly, the group that purchases PHI is at a higher economic level and thus less prone to fall into CHE and impoverishment. Our further analyses, using the same data, showed that the richest households (5th quintiles) had the highest coverage rate of PHI compared to other quintiles in 2015 and 2017, providing a concrete evidence to confirm the point mentioned (supplemental Tables 1 and supplemental Table 2). Secondly, the low level of benefit packages from basic medical insurance is insufficient to cope with the rapid increase in medical costs, as confirmed by several existing studies finding in China [9–11].

We compared the degree of protection for financial risk between RBMI + PHI and RBMI alone. As shown in Figs. 1 and 2, RBMI + PHI was more effective in preventing CHE and impoverishment than RBMI alone, without considering control variables. Furthermore, random effects panel logit regression model was conducted to further verify the effect of PHI on financial catastrophe and impoverishment due to health care spending. After controlling a series of variables related to the characteristics of each household and its household head, we found that RBMI + PHI could further significantly reduce the CHE incidence (5.4 %) and impoverishment rate (4.9 %) compared with RBMI alone. In order to address endogeneity problems, PSM method was employed to eliminate the effect of self-selection on the estimation results. As expected, the results were still robust after using PSM method. In other words, PHI further played an effective role in protecting financial risks of households in the context of universal basic medical insurance coverage. In theory, the underlying mechanism for the above phenomenon is that PHI could reduce the out-of-pocket medical expenditure to alleviate the financial risks of households with patients. Our further analysis using individual-level data indicated that the out-of-pocket inpatient expenditure as a percentage of total inpatient expenditure for individuals covered by RBMI + PHI was lower than that for individuals covered by RBMI alone (2015: 46.09 % vs. 62.14 %, 2017: 53.51 % vs. 59.48 %).

With respect to the effect of PHI on alleviating financial risks associated with diseases, previous studies using data from the U.S. showed similar conclusions to this study [48, 49]. However, the necessity of this study lies in the fact that there are significant differences between the national conditions of the U.S. and China. Firstly, PHI plays an important role in the U.S. medical insurance system, while it only plays an auxiliary role in the medical insurance system of China. Secondly, the development of PHI in the U.S. has been relatively mature, but the development of PHI in China is still in its infancy. Our findings suggested that PHI has initially achieved the main purpose of reducing the economic risk associated to illness.

Additionally, this study showed that several key determinants, such as household size, receiving inpatient services, having members over 60 years old, economic status, geographic location, residency location, education, employment status, and self-rated health status, exerted an impact on economic protection, as reported in previous studies [28, 29, 50]. This study only regarded these influencing factors as control variables without detailed explanation.

Several limitations must be noted here. Firstly, a conservative method was employed to calculate the out-of-pocket medical expenditure. Specifically, indirect expenditures (e.g., accommodation cost, food, lost productivity due to illness, etc.) were not included in out-of-pocket medical expenses, leading to underestimation of the CHE incidence to some extent [51]. Secondly, the database did not capture specific information on disease types (e.g., hypertension, dyslipidemia, diabetes, chronic lung disease, disability, etc.). Hence, this study could not specifically analyze households suffering from a certain disease. Thirdly, PSM method could only solve the problem of self-selection by matching the control group to the treatment group based on the observable variables. Therefore, the ATT could not reflect the effect of unobservable variables on financial risk protection [52]. In the further studies, the combination of DID and PSM may be employed to eliminate the effect of unobservable variables.

## Conclusions

In summary, high incidences of CHE and impoverishment after reimbursement from medical insurance were found among Chinese households with RBMI in both years. Households reimbursed from RBMI + PHI had lower incidences of CHE and impoverishment due to health spending than households reimbursed from RBMI alone. PHI played an effective role in protecting financial risks of households in the context of universal basic medical insurance coverage.

## Supplementary Information

**Additional file 1: **

## Data Availability

Data and materials used during the current study are publicly available on the CHFS official website (see link: https://chfs.swufe.edu.cn/datacenter/apply.html).
