# Supplementary material for: Financial protection effects of private health insurance: experimental evidence from Chinese households with resident basic medical insurance
Source: Int J Equity Health. 2021 May 17;20:122. doi: 10.1186/s12939-021-01468-5 (PMC8130397; doi:10.1186/s12939-021-01468-5)
Supplement: Supplementary file 1 — Additional file 1: [file 12939_2021_1468_MOESM1_ESM.docx]

**Supplemental Table 1.** Number of households reimbursed from RBMI plus PHI, China, 2015.

| Economic status | Description | Total | RBMI plus PHI | RBMI alone |
| --- | --- | --- | --- | --- |
| Quintile 1, n (%) | 20% low income households | 3630 (100.00) | 31 (0.85) | 3599 (99.15) |
| Quintile 2, n (%) | 20% low middle income households | 3630 (100.00) | 33 (0.91) | 3597 (99.09) |
| Quintile 3, n (%) | 20% middle income households | 3629 (100.00) | 58 (1.60) | 3571 (98.40) |
| Quintile 4, n (%) | 20% high middle income households | 3629 (100.00) | 98 (2.70) | 3531 (97.30) |
| Quintile 5, n (%) | 20% high income households | 3631 (100.00) | 199 (5.48) | 3432 (94.52) |

Note: *RBMI* Resident basic medical insurance; *PHI* Private health insurance.

**Supplemental Table 2.** Number of households reimbursed from RBMI plus PHI, China, 2017.

| Economic status | Description | Total | RBMI plus PHI | RBMI alone |
| --- | --- | --- | --- | --- |
| Quintile 1, n (%) | 20% low income households | 3631 (100.00) | 95 (2.62) | 3536 (97.38) |
| Quintile 2, n (%) | 20% low middle income households | 3629 (100.00) | 144 (3.97) | 3485 (96.03) |
| Quintile 3, n (%) | 20% middle income households | 3630 (100.00) | 200 (5.51) | 3430 (94.49) |
| Quintile 4, n (%) | 20% high middle income households | 3629 (100.00) | 324 (8.93) | 3305 (91.07) |
| Quintile 5, n (%) | 20% high income households | 3630 (100.00) | 636 (17.52) | 2994 (82.48) |

Note: *RBMI* Resident basic medical insurance; *PHI* Private health insurance.
